# Static Tactile Sensing Based on Electrospun Piezoelectric Nanofiber Membrane

**DOI:** 10.3390/s22186779

**Published:** 2022-09-08

**Authors:** Hyunjung Cho, Taejoon Kouh

**Affiliations:** Department of Physics, Kookmin University, Seoul 136-702, Korea

**Keywords:** static tactile sensor, electrospun nanofiber, piezoelectricity, mechanical oscillation

## Abstract

Here, a static tactile sensing scheme based on a piezoelectric nanofiber membrane, prepared via the electrospinning method, is presented. When the nanofiber membrane is kept under a constant vibration, an external contact onto the membrane will attenuate its vibration. By monitoring this change in the oscillation amplitude due to the physical contact via the piezoelectrically coupled voltage from the nanofiber membrane, the strength and duration of the static contact can be determined. The proof-of-concept experiment demonstrated here shows that the realization of a static tactile sensor is possible by implementing the piezoelectric nanofiber membrane as an effective sensing element.

## 1. Introduction

A tactile sensor is a device or system that can convert an external stimulus—especially in the form of physical contact or pressure—into an electrical output signal [1,2,3]. This has been utilized in many industrial applications, and further uses can be expected in the emerging technological fields such as artificial skin, robotic arms, or minimally invasive surgery [4,5,6,7,8,9,10,11,12,13,14,15,16,17]. Various types of tactile sensors have been developed so far based on capacitive, piezoelectric, piezoresistive, or optical sensing techniques [18,19,20,21,22,23,24,25].

Some of the key factors to be considered in the development of a tactile sensor are flexibility, durability—often associated with the flexibility—and its sensitivity. Considering these, piezoelectric polymers such as polyvinylidene fluoride (PVDF) are found to be excellent candidate materials for the development of a tactile sensor [26,27,28,29,30]. These are highly flexible and strong against physical impact. Moreover, studies show that they can offer high piezoelectricity, which can effectively transduce the mechanical load to the electric signal [31,32,33,34,35].

The piezoelectric effect in these sensors arises from the instantaneous polarization of the polymer chains due to mechanical deformation such as stretching or bending. It is coupled to strain velocity; therefore, when the deformation remains constant, the polymer chains can no longer maintain their piezoelectricity [36,37,38]. The presence of the polarized charges can be continuously stimulated by time-varying repeated deformation, which makes the development of dynamic tactile sensor feasible, but, for the case of the static deformation—where the external load remains constant over a long period of time—the rapid disappearance of the polarization cannot guarantee the piezoelectric effect long enough to detect any static deformation. This intrinsic limit prevents the application of the piezoelectric polymer as a static tactile sensor element. Hence, many of the development strategies for static tactile sensors based on PVDF are limited to the quasi-static sensing, or are required to incorporate other mechanisms such as capacitive and piezoresistive effects, using various PVDF-based composites or complex sensing structures.

**Figure 1:**
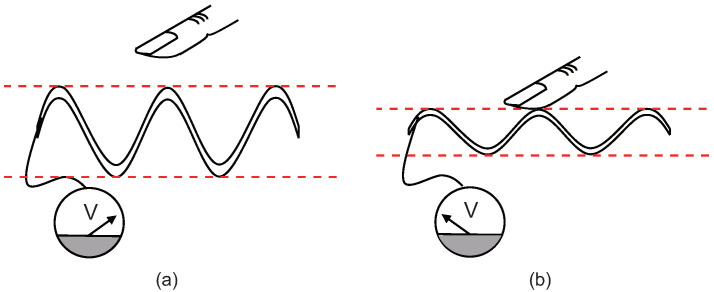
Proposed static tactile sensing method. (**a**) When no external contact is made, the piezoelectric membrane, driven oscillating with constant amplitude, generates the voltage signal. (**b**) When external contact is made, this will suppress the vibration amplitude of the membrane, resulting in a smaller piezoelectric voltage signal compared to the value at contact-free oscillation. By monitoring this change in the piezoelectric output signal, the presence of the external contact can be detected.

In this paper, we demonstrate a static tactile sensing technique solely based on the piezoelectric polymer membrane, made from electrospun nanofibers. The key concept in this method is that the polymer membrane is intentionally set to vibrate with a predetermined vibration amplitude and frequency by an external source, and its vibration amplitude is measured continuously via the piezoelectric effect. Since the mechanical load brought into the close proximity of the oscillating membrane is expected to suppress its oscillation amplitude—and, correspondingly, the piezoelectric signal from the membrane—as long as the load remains on the membrane, this sensing scheme will allow the identification of the presence of the steady physical contact, as well as its duration (Figure 1). The proposed static sensing method, as presented here, can offer a simple means of developing a static tactile sensor using a flexible piezoelectric polymer material, without the need of composite polymer materials or additional design considerations in the sensor element.

## 2. Materials and Methods

Regarding the piezoelectric polymer, a thin PVDF nanofiber membrane was fabricated via an electrospinning process. Prior to the electrospinning process, a sol–gel mixture was prepared by adding N,N-Dimethylformamide (DMF, Sigma Aldrich, St. Louis, MI, USA) and Acetone (Duksan General Science, Seoul, Korea), mixed in a volume ratio of 2:1 with PVDF (molar mass of 275,000 g/mol, Sigma-Aldrich) as a solute. Finally, 25 wt.% PVDF solution was stirred at 70 ${}^{\circ }C$ for 4 h to produce a homogeneous solution for bead-free PVDF nanofibers.

**Figure 2:**
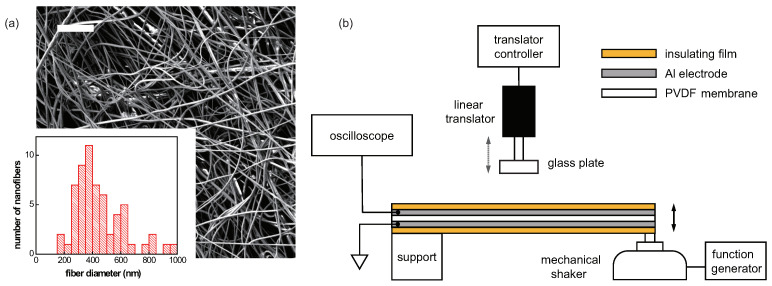
(**a**) Scanning electron micrograph of the electrospun PVDF nanofibers. (The scale bar on the top left corner is 10 μm.) The lower inset shows the histogram of the nanofiber diameter distribution with a mean value around 450 nm. (**b**) Illustration of the experimental setup. The sensing element is composed of the piezoelectric PVDF membrane, sandwiched between Al electrodes and protective insulating films. One end of the sensing element is fixed on a support structure and the other end is connected to a mechanical shaker. While the membrane is driven to oscillation, the resulting piezoelectric voltage is monitored with an oscilloscope. To observe the piezoelectric response of the membrane under the static external load, the mechanical contact is established by approaching the glass plate, attached to a linear translator, onto the PVDF membrane.

The prepared mixture solution was electrospun using a single-nozzle electrospinning machine (Inovenso NE200). PVDF solution was loaded into a 10-mL plastic syringe and a 22-gauge needle was connected to its end. A thin layer of nanofibers was produced at 16 kV with a tip-to-collector distance of 15 cm, with the syringe pump pushing the solution at a constant rate of 2 mL/h. The high electric field during the electrospinning process can induce a high content of β phase, responsible for its piezoelectric property, in a PVDF nanofiber structure through the local poling effect [39]. Moreover, since high humidity is important to obtain a β-rich phase in PVDF nanofibers [40], the humidity level was kept around 60% during the process. To ensure that the fabricated PVDF nanofibers were bead-free, the morphology of the electrospun fibers was also examined with a field-emission scanning electron microscope (Jeol JSM-7401F). The scanning electron micrograph of the fabricated nanofibers showed that they were free of beads, with a mean diameter of ∼450 nm. (Figure 2a) As a last step, the nanofiber membrane collected on an Al foil was peeled off and placed between two Al electrodes.

To experimentally observe the piezoelectric response of the PVDF nanofiber membrane upon mechanical contact, the sensing element is formed by encapsulating the membrane with two Al electrodes, as well as protective insulating polyimide films both on top and bottom. The total thickness of this sensing element is around 330 μm, with the thickness of the membrane around 20 μm. It is clamped at one end, and the other end is fixed onto a mechanical shaker (Segye SG-YS5426), as illustrated in Figure 2b. The piezoelectric voltage across the electrodes is measured with an oscilloscope (Tektronix TDS3032), while driving the membrane into vibration with the mechanical shaker. The vibration amplitude and the frequency are controlled by a function generator (Tektronix AFG3021B), connected to the shaker.

For the further demonstration of the static sensing based on the PVDF polymer, the physical load is simulated by bringing in a small piece of glass plate, attached to a linear translator, onto a vibrating membrane, while the piezoelectric voltage is continuously monitored and compared to the output signal during the contact-free oscillation of the membrane. In our proof-of-concept experiment, the level of the mechanical contact is given by the separation between the membrane and the glass plate, and the separation between them is varied by the motion of the linear translator, controlled by a motion controller (Micronix MMC-100).

## 3. Results and Discussion

**Figure 3:**
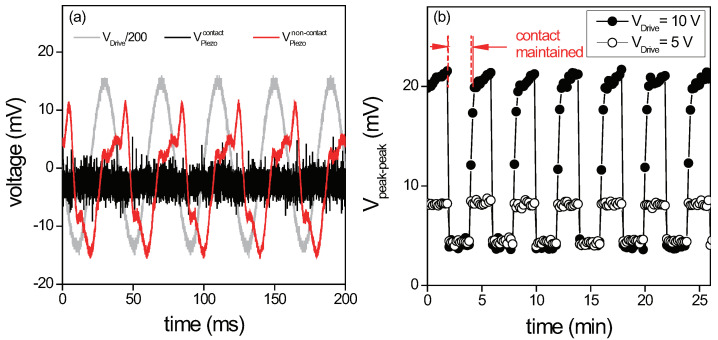
(**a**) When no external contact is made, the freely oscillating PVDF membrane, driven with constant amplitude, generates a significant piezoelectric voltage signal $V_{Piezo}^{non-contact}$ at the frequency of the driving signal $V_{Drive}$. However, once the contact is established and prevents the membrane from the continuous vibration, no periodic piezoelectric signal $V_{Piezo}^{contact}$ is detected. For comparison with the piezoelectric signal, the driving voltage is divided by a factor of 200. (**b**) Peak–to–peak piezoelectric voltage signal $V_{peak-peak}$ at 25 Hz, measured under the repeated application of the mechanical contact, for two different drive voltages of 5 and 10 V. Without contact for a period of 120 s, the large constant piezoelectric signal can be measured. However, for the contact duration of 120 s, only the voltage at the level of the electrical noise is detected. When this measurement is performed over 26 min, the output signal curve shows constant switching between the contact and non-contact at each repeated cycle.

The piezoelectric voltage response of the PVDF nanofiber membrane is displayed in Figure 3a, while being driven by a mechanical shaker at the driving frequency of 25 Hz. When there is no physical contact made onto the membrane, the measured piezoelectric voltage $V_{Piezo}^{non-contact}$ across the pair of Al electrodes shows a large piezoelectric response due to the continuous mechanical deformation of the PVDF membrane, clearly matching the driving frequency of 25 Hz. This confirms the expected piezoelectric characteristics of the prepared nanofiber membrane.

Later, a glass plate is brought onto the membrane surface to obtain tight contact between them. Since this suppresses the oscillatory motion of the membrane without any room for vibration, the piezoelectric response is no longer present in the measured output voltage curve of $V_{Piezo}^{contact}$, as shown in Figure 3a. This clearly indicates that the proposed tactile sensing scheme based on the piezoelectric polymer membrane can be used to detect the presence of external contact.

To achieve true static sensing, the sensing element must be able to generate and maintain the output signal for a long period of time, regardless of the presence of the contact. To demonstrate this, the peak-to-peak value of the output voltage is measured continuously while the glass plate is firmly pushed onto or pulled away from the membrane surface repeatedly using the linear translator. The contact is made and maintained for 120 s, and the contact is removed from the surface for the following 120 s. Figure 3b shows the repeated measurements performed for total of 26 min. When the contact is made, the sensor returns a small voltage value of ∼5 mV, the value corresponding to the electrical noise as seen in Figure 3a, but when the contact is removed and the membrane oscillates freely, it generates the peak-to-peak output value of ∼25 mV. For each attempt, the sensing element is able to maintain the output signal and this allows the detection of the presence and duration of the static contact for a long period of time, as expected. When the same measurement is attempted for a smaller drive voltage, the response of the PVDF membrane is identical, but with a smaller piezoelectric voltage output.

**Figure 4:**
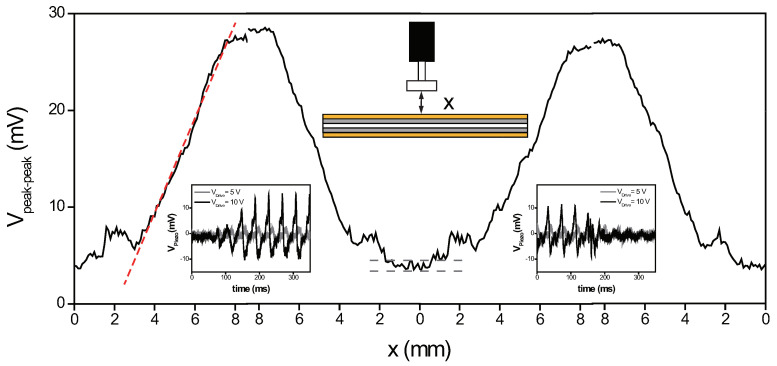
Tactile sensing response as a function of the contact separation. The piezoelectric peak–to–peak voltage $V_{peak-peak}$ is monitored while the contact distance *x* between the sensing element and the glass plate is varied. Between the loading (x=0 mm) and unloading (x=8 mm) of the physical contact, a linear increase and decrease in the sensing signal can be observed. When repeated, the sensing element shows a steady response. The red dotted line is a linear fit with a slope of 4.9 mV/mm. In addition, a pair of gray dotted lines in the middle indicates the measurement uncertainty (∼1 mV) in this experiment, which results in the minimum detectable contact separation around 200 μm. The insets show the time-transient curves when the contact is removed (inset on left) and when the contact is made (inset on right) under two different drive voltages of 5 and 10 V.

The response from the vibrating piezoelectric PVDF membrane is also investigated as a function of the level of the contact by varying the separation between the membrane and the glass plate attached on the linear translator. This can provide an insight into the sensitivity of the static sensing scheme in terms of the proximity of the contact (Figure 4). The glass plate is initially pressed onto the membrane surface tightly, resulting in no piezoelectric voltage signal. As the glass plate is pulled away, one can observe a slow increase in the output signal. Once the glass plate is fully separated from the membrane, around the separation distance near 8 mm, the output signal due to the contact-free oscillatory motion reaches its maximum value. If the motion of the linear translator is reversed and the glass plate is moved toward the sensing element, the voltage signal slowly decreases from its maximum value and drops back to the minimum value because of the full physical contact.

When repeated, the sensing element can generate a reliable and steady response depending on how close the contact to the membrane. The incremental increase and decrease in the output signal show a linear change with the contact separation. The change in the piezoelectric output signal is least squared fitted to a straight line, with a goodness-of-fit, given by the adjusted R-squared, of 0.98685. From the slope of this fitted linear curve, we can estimate the sensitivity of this proof-of concept experiment with respect to the contact separation, which is approximately 4.9 mV/mm. Moreover, by considering the uncertainty in voltage measurement, which is around a peak-to-peak value of 1 mV, the minimum detectable contact separation would be approximately 200 μm, even in this simple proof-of-concept experiment. We have also measured the time-transient curves when the contact is removed or when the contact is made, as shown in the two insets in Figure 4. These curves show that the response time upon the contact, in which the output signal changes from maximum to minimum or from minimum to maximum, is estimated to be around ∼100 ms on average, ensuring a fast response to capture the mechanical deformation caused by the physical contact.

## 4. Conclusions

The proof-of-concept experiment presented here illustrates that static tactile sensing is feasible based on the driven oscillation of the piezoelectric membrane, while the typical implementation of the piezoelectric polymer has been limited to the dynamic-only detection. Our work clearly opens up new opportunities in the further integration of the piezoelectric polymer in tactile sensor applications.

However, there is also room for improvement beyond the experiment demonstrated here. The effectiveness of the sensing scheme can benefit from flexible polymer materials with higher piezoelectric properties, which will generate a higher response to the external stimulus. Moreover, additional careful design considerations would be desirable. For instance, one can notice the consistent appearance of small bumps in the contact separation-versus-piezoelectric voltage curve in Figure 4, when the contact is initialized or breaks at close proximity to the membrane. Thus far, it is not clear why these small deviations from the expected linear sensor response occur. It might be due to the surface interaction between the contact glass and the polyimide insulating film, such as an adhesion effect; the issue of the rigidity of the sensing element as it is strongly pulled or pushed—especially when close physical contact is established—or simply insufficient clamping of both ends of the membrane. Fine and detailed optimization of the sensor parameters will certainly lead to the more reliable operation of the static tactile sensing scheme presented.

## Data Availability

The data presented in this study are available upon reasonable request from the authors.

## Abbreviations

The following abbreviations are used in this manuscript:

PVDF	Polyvinylidene fluoride
DMF	N,N-Dimethylformamide

## References

[1] Yu P., Liu W., Gu C., Cheng X., Fu X. (2016). Flexible Piezoelectric Tactile Sensor Array for Dynamic Three-Axis Force Measurement. Sensors.

[2] Li C., Wu P., Lee S., Gorton A., Schulz M.J., Ahn C.H. (2008). Flexible Dome and Bump Shape Piezoelectric Tactile Sensors Using PVDF-TrFE Copolymer. J. Microelectromech. Syst..

[3] Dargahi J., Parameswaran M., Payandeh S. (2000). A micromachined piezoelectric tactile sensor for an endoscopic grasper-theory, fabrication and experiments. J. Microelectromech. Syst..

[4] Sun X., Sun J., Zheng S., Wang C., Tan W., Zhang J., Liu C., Liu C., Li T., Qi Z. (2019). A Sensitive Piezoresistive Tactile Sensor Combining Two Microstructures. Nanomaterials.

[5] Lucarotti C., Oddo C.M., Vitiello N., Carrozza M.C. (2013). Synthetic and Bio-Artificial Tactile Sensing: A Review. Sensors.

[6] Seminara L., Pinna L., Valle M., Basiricò L., Loi A., Cosseddu P., Bonfiglio A., Ascia A., Biso M., Ansaldo A. (2013). Piezoelectric Polymer Transducer Arrays for Flexible Tactile Sensors. IEEE Sens. J..

[7] Kim M.-S., Ahn H.-R., Lee S., Kim C., Kim Y.-J. (2014). A dome-shaped piezoelectric tactile sensor arrays fabricated by an air inflation technique. Sens. Actuator A Phys..

[8] Girão P.S., Ramos P.M.P., Postolache O., Pereira J.M.D. (2013). Tactile sensors for robotic applications. Measurement.

[9] Hwang S.K., Hwang H.Y. (2013). Development of a tactile sensing system using piezoelectric robot skin materials. Measurement.

[10] Kimoto A., Sugitani N., Fujisaki S. (2010). A Multifunctional Tactile Sensor Based on PVDF Films for Identification of Materials. IEEE Sens. J..

[11] Chen S., Jiang K., Lou Z., Chen D., Shen G. (2018). Recent Developments in Graphene-Based Tactile Sensors and E-Skins. Adv. Mater. Technol..

[12] Pyo S., Lee J., Bae K., Sim S., Kim J. (2021). Recent Progress in Flexible Tactile Sensors for Human-Interactive Systems: From Sensors to Advanced Applications. Adv. Mater..

[13] Lee M.H., Nicholls H.R. (1999). Review Article Tactile Sensing for Mechatronics—A State of the Art Survey. Mechatronics.

[14] Zou L., Ge C., Wang Z., Cretu E., Li X. (2017). Novel Tactile Sensor Technology and Smart Tactile Sensing Systems: A Review. Sensors.

[15] Puangmali P., Althoefer K., Seneviratne L.D., Murphy D., Dasgupta P. (2008). State-of-the-Art in Force and Tactile Sensing for Minimally Invasive Surgery. IEEE Sens. J..

[16] Zhang Y., Ju F., Wei X., Wang D., Wang Y. (2020). A Piezoelectric Tactile Sensor for Tissue Stiffness Detection with Arbitrary Contact Angle. Sensors.

[17] Sokhanvar S., Packirisamy M., Dargahi J. (2007). A Multifunctional PVDF-Based Tactile Sensor for Minimally Invasive Surgery. Smart Mater. Struct..

[18] Tiwana M.I., Redmond S.J., Lovell N.H. (2012). A review of tactile sensing technologies with applications in biomedical engineering. Sens. Actuator A Phys..

[19] Kim H.-K., Lee S., Yun K.-S. (2011). Capacitive Tactile Sensor Array for Touch Screen Application. Sens. Actuators A Phys..

[20] Li T., Luo H., Qin L., Wang X., Xiong Z., Ding H., Gu Y., Liu Z., Zhang T. (2016). Flexible Capacitive Tactile Sensor Based on Micropatterned Dielectric Layer. Small.

[21] Stassi S., Cauda V., Canavese G., Pirri C. (2014). Flexible Tactile Sensing Based on Piezoresistive Composites: A Review. Sensors.

[22] Kane B.J., Cutkosky M.R., Kovacs G.T.A. (1996). CMOS-Compatible Traction Stress Sensor for Use in High-Resolution Tactile Imaging. Sens. Actuators A Phys..

[23] Nguyen T.-D., Lee J.S. (2021). Recent Development of Flexible Tactile Sensors and Their Applications. Sensors.

[24] Long Z., Liu X., Xu J., Huang Y., Wang Z. (2022). High-Sensitivity Flexible Piezoresistive Pressure Sensor Using PDMS/MWNTS Nanocomposite Membrane Reinforced with Isopropanol for Pulse Detection. Sensors.

[25] Duan Z., Jiang Y., Huang Q., Yuan Z., Zhao Q., Wang S., Zhang Y., Tai H. (2021). A do-it-yourself approach to achieving a flexible pressure sensor using daily use materials. J. Mater. Chem. C.

[26] Park J., Kim M., Lee Y., Lee H.S., Ko H. (2015). Fingertip skin-inspired microstructured ferroelectric skins discriminate static/dynamic pressure and temperature stimuli. Sci. Adv..

[27] Navaraj W., Dahiya R. (2019). Fingerprint-Enhanced Capacitive-Piezoelectric Flexible Sensing Skin to Discriminate Static and Dynamic Tactile Stimuli. Adv. Intell. Syst..

[28] Fastier-Wooller J.W., Vu T.-H., Nguyen H., Nguyen H.-Q., Rybachuk M., Zhu Y., Dao D.V., Dau V.T. (2022). Multimodal Fibrous Static and Dynamic Tactile Sensor. ACS Appl. Mater. Interfaces.

[29] Lin W., Wang B., Peng G., Shan Y., Hu H., Yang Z. (2021). Skin-Inspired Piezoelectric Tactile Sensor Array with Crosstalk-Free Row+Column Electrodes for Spatiotemporally Distinguishing Diverse Stimuli. Adv. Sci..

[30] Jiang J., Tu S., Fu R., Li J., Hu F., Yan B., Gu Y., Chen S. (2020). Flexible Piezoelectric Pressure Tactile Sensor Based on Electrospun BaTiO_3_/Poly(vinylidene fluoride) Nanocomposite Membrane. ACS Appl. Mater. Interfaces.

[31] Sabouni Tabari R., Chen Y., Thummavichai K., Zhang Y., Saadi Z., Neves A.I.S., Xia Y., Zhu Y. (2022). Piezoelectric Property of Electrospun PVDF Nanofibers as Linking Tips of Artificial-Hair-Cell Structures in Cochlea. Nanomaterials.

[32] Kawai H. (1969). The Piezoelectricity of Poly (vinylidene Fluoride). Jpn. J. Appl. Phys..

[33] Lu L., Ding W., Liu J., Yang B. (2020). Flexible PVDF Based Piezoelectric Nanogenerators. Nano Energy.

[34] Wang Y.R., Zheng J.M., Ren G.Y., Zhang P.H., Xu C. (2011). A Flexible Piezoelectric Force Sensor Based on PVDF Fabrics. Smart Mater. Struct..

[35] Kalimuldina G., Turdakyn N., Abay I., Medeubayev A., Nurpeissova A., Adair D., Bakenov Z. (2020). A Review of Piezoelectric PVDF Film by Electrospinning and Its Applications. Sensors.

[36] Yuji J.-I., Sonoda C. A PVDF Tactile Sensor for Static Contact Force and Contact Temperature. Proceedings of the 2006 5th IEEE Conference on Sensors.

[37] Dargahi J., Kahrizi M., Purushotham Rao N., Sokhanvar S. (2006). Design and Microfabrication of a Hybrid Piezoelectric-capacitive Tactile Sensor. Sens. Rev..

[38] Wang S., Shao H.-Q., Liu Y., Tang C.-Y., Zhao X., Ke K., Bao R.-Y., Yang M.-B., Yang W. (2021). Boosting Piezoelectric Response of PVDF-TrFE via MXene for Self-Powered Linear Pressure Sensor. Compos. Sci. Technol..

[39] Gade H., Nikam S., Chase G.G., Reneker D.H. (2021). Effect of Electrospinning Conditions on *β*-Phase and Surface Charge Potential of PVDF Fibers. Polymer.

[40] He Z., Rault F., Lewandowski M., Mohsenzadeh E., Salaun F. (2021). Electrospun PVDF Nanofibers for Piezoelectric Applications: A Review of the Influence of Electrospinning Parameters on the *β* Phase and Crystallinity Enhancement. Polymers.

